# A study on the relationship between job stress and nicotine dependence in Korean workers

**DOI:** 10.1186/s40557-016-0113-4

**Published:** 2016-06-10

**Authors:** Seung Rak Son, Byeong Moo Choe, Seong Hwan Kim, Young Seoub Hong, Byoung Gwon Kim

**Affiliations:** Department of Psychiatry, College of Medicine, Dong-A University, Busan, Republic of Korea; Department of Preventive Medicine, College of Medicine, Dong-A University, Busan, Republic of Korea; Regional Cardiocerebrovascular Center, Dong-A University Medical Center, Busan, Republic of Korea

**Keywords:** Nicotine dependence, Job stress, Smoking, Workers

## Abstract

**Background:**

Nicotine dependence and its severity are often determined by individuals’ psychosocial factors.This study purposed to analyze how Korean workers’ job stress is related with their nicotine dependence according to demographic and occupational characteristics in order to reduce smoking related to job stress and to establish objective indicators to be used in developing adequate smoking cessation strategies.

**Methods:**

The subjects of this study were 4,639 workers who replied to the questionnaire survey. In addition, 1,948 current smokers were separated from non-smokers and ex-smokers, and the relationship between job stress and nicotine dependence was analyzed with the current smoker group. Nicotine dependence was tested using Fagerström’s Test of Nicotine Dependence, and stress was measured using a questionnaire on subjective stress felt by workers in their daily life and the short form of the Korean Occupational Stress Scale.

**Results:**

The smoking rate was 54.1 % among men and 2.5 % among women. Nicotine dependence was significantly different according to interpersonal conflict, organization system and lack of reward (*p* < 0.05). As multivariate logistic analysis, job control, occupational climate and total stress score were statistical significant (*p* < 0.05).

**Conclusions:**

Job stress was found to be related with smoking status and nicotine dependence. Based on this result, it is suggested to enhance workers’ welfare for health promotion in workplace by improving their working environment in order to reduce job stress and consequently to decrease the smoking rate.

## Background

Smoking is known to be a major and the most preventable cause of a large variety of cancers, heart diseases, apoplexy, pregnancy complications, chronic obstructive pulmonary diseases and early death. Due to the high smoking rate in adult men and the increasing smoking rate among adolescents, smoking cessation has become an immediate concern for raising Korean people’s health level. In this situation, many associations and organizations are conducting smoking cessation education and anti-smoking campaigns and there are active movements for creating anti-smoking environment such as regulations on cigarette advertisements, warning signs against smoking, designation of non-smoking areas in public places, and prohibition of cigarette sale to adolescents [1].

On the other hand, the reason for failure of smoking cessation in smokers is their dependence on nicotine contained in cigarette. When they inhale cigarette smoke, nicotine is transmitted deep into the lungs and absorbed rapidly into the blood vessels. Nicotine absorbed into the body has an arousal effect, increases attention, and makes the person feel pleasant. These effects last only temporarily and smokers have to keep smoking in order to maintain such effects continuously. What is more, if nicotine is absorbed into the body continuously the body comes to have tolerance to such effects and the smoking quantity increases in order to attain effects of the same intensity. Therefore, continuous smoking results in increased nicotine dependence, and such dependence is manifested in two types, namely, physical dependence and psychological dependence. First, physical dependence means that smokers have tolerance and experience withdrawal symptoms caused by nicotine deficiency whenever they try to stop smoking and such symptoms include depression, insomnia, anxiety, low attention, headache, and fatigue [2]. In addition, psychological dependence occurs when the smoker begins to associate a feeling, situation or environmental stimulus with the compensation effect of nicotine. That is, if one associates smoking with relief from anxiety or stress, he/she is simulated to smoke whenever he/she feels anxiety or stress, and if one associates smoking with increased pleasure, he/she has a strong desire to smoke during social activities, after a meal, or while drinking. In other words, smoking is involved deeply in the individual’s life and smoking behavior is automated in daily life [3]. Moreover, nicotine dependence and its severity are often determined by individuals’ psychosocial factors [4]. In particular, job stress is known to be a psychosocial factor having a considerable effect on the start and extent of smoking [5, 6]. Nicotine dependence can be tested by various methods but the most common method is Fagerström’s Test for Nicotine Dependence (FTND) consisting of 6 questions. This test is also used to predict success in smoking cessation or to determine the replacement dose of nicotine. In addition, FTND is a statistically proven instrument for evaluating the severity of nicotine dependence, and because it has been used in many other studies the results of evaluation can be compared with previous reports.

Job stress has been reported to cause not only physical diseases but also behavioral changes and psychological problems. That is, it is reported to induce physical and mental diseases as well as changes in individuals’ behavioral pattern such as increased smoking, drug dependence, caffeine intake, and alcohol intake [7–9]. Employment insecurity and the flexibility of the labor market resulting from recent economic changes are expected to aggravate wage workers’ job stress and its adverse effects on health.

Thus, this study purposed to analyze how Korean workers’ job stress is related with nicotine dependence according to demographic and occupational characteristics in order to reduce smoking related to job stress and to establish objective indicators to be used in developing adequate smoking cessation strategies.

## Methods

### Study population

The subjects of this study were sampled from workplaces throughout nation-wide, and a questionnaire survey was conducted. Initially, we designed that the distribution of workers, as 10,000 of participants, was fitted for national proportions of company size in Korea. The workplaces were selected at random in consideration of the distribution of workplaces below 49 employees, those with 50–99, those with 100–299, and those above 300, and accessibility to the workplaces. In order to distribute questionnaires evenly according to industry size, we limited the number of workers to be surveyed so that the sample size of each industry size would not exceed 20 % of the total sample size. In addition, sampling was made evenly among surveyed areas and business categories. Among the workers who replied to the questionnaire survey, those who answered all the questions were analyzed for their demographic and occupational characteristics and their smoking status. In addition, current smokers were separated from non-smokers and ex-smokers, and the relationship between job stress and nicotine dependence was analyzed with the current smoker group.

### Questionnaire survey

For the workers who participated in the survey, we explained the purposes and contents of the survey and obtained their written consent to participate in the survey voluntarily. Questionnaires having questions not answered by the subjects were excluded from analysis.

Considering the first return rate will be lower than planned, we distributed 2,000 questionnaires additionally. As a result, a total of 12,000 questionnaires were distributed and 8,466 of them were returned, so the overall return rate was 70.5 % and the recovery rate based on the initial plan was 84.7 %. In addition, 561 workplaces participated and the return rate in terms of workplaces was 56.1 % based on the initial plan.

We conducted resampling for adjusting the national proportions for company size. Among the subjects who answered the survey, 4,639 workers replied all the questions without omission and they were used as the valid sample in analyzing the relationships among demographic and occupational characteristics, smoking status, subjective stress and job stress. In addition, 1,921 current smokers excepting female were separated from non-smokers and ex-smokers, and the relationship between job stress and nicotine dependence was analyzed with the current smoker group.

### Contents of the questionnaire

General demographic characteristics surveyed were age, gender, marital status and academic qualification, and job-related characteristics were weekly working hour, whether to have breaks, employment type, job type, industry type, and position. Academic qualification was divided into middle school or lower, high school, college, and graduate school, and marital status was divided into single, married, and divorced/bereaved. Employment type was divided into regular workers, contract workers, and part-time/temporary workers, and job type was divided largely into blue-collar workers and white-collar workers. Position was divided into rank-and-file workers, assistant managers/deputy managers, and managers.

Questions on smoking asked about basic smoking characteristics such as current smoking status, the age of the first smoking, smoking period, daily smoking quantity, and whether to have attempted smoking cessation. For ex-smokers, smoking period, average smoking quantity, and period after smoking cessation were surveyed additionally. For current smokers, nicotine dependence was measured using FTND [10] and the obtained score was graded into low dependence (0–5 point) and high dependence (6–10).

For stress, we surveyed subjective stress that the workers felt in daily life, and job stress that they felt in workplace. Subjective stress felt in daily life was measured using the Subjective Stress Scale used in the National Health and Nutrition Survey. Job stress was evaluated using the short form of the Korean Occupational Stress Scale developed by Jang et al. [11], which is a standardized instrument whose reliability and validity were tested with 30,146 Korean workers sampled throughout the country and focuses on measuring wage workers’ job stress factors, and this tool was chosen in order to identify the causes of job stress specific to Korean environments and Korean workers. While the basic form of this questionnaire consisted of 43 questions, this study used its short form containing 24 questions selected based on the internal consistency and discriminant validity of questions and through factor analysis. The questions were divided into job stress factors such as job demand, lack of job control, interpersonal conflict, job insecurity, organization system, lack of reward, and occupational climate. Each question was answered ‘Absolutely not,’ ‘No,’ ‘Yes’ and ‘Absolutely yes.’ The answers were encoded on a 1-to-4-point scale if a high score meant high job stress or on a 4-to-1-point scale if a high score meant low job stress, and then the total score of each area was calculated and it was converted to a score out of 100. The total score of job stress was obtained by summing up the scores of the 7 factors and dividing the sum by 7. The individuals were divided into quartile groups according to their job stress score based on Korean workers’ quartiles by gender, and their job stress level were compared according to each factor.

### Statistical analyses

This study conducted cross tabulation analysis on demographic and occupational characteristics and smoking status. In addition, cross tabulation analysis was used in order to compare nicotine dependence, subjective stress and job stress according to demographic and occupational characteristics and smoking status. Furthermore, student t-test was performed in order to compare the mean job stress according to demographic and occupational characteristics or according to nicotine dependence. The significance level was 5 % (*p* < 0.05) in each test, and STATA/SE 12.0 (StataCorp., College Station, TX, USA) was used in all the statistical analyses. Univariate and multivariate logistic analysis were conducted by adjusting age, education marital status and job-type. The ethical issues of the study were approved by the Institutional Review Board of Dong-A University Hospital (ID: DAUHIRB-EXP-16-066).

## Results

### General characteristics of study population

As to the distribution of the subjects’ demographic characteristics, the age group of 30–39 was largest (42.4 %), followed by the group of 29 or younger (28.6 %). According to gender, 76.5 % were male. According to education level, most of the subjects were high school or college (91.5 %), and according to marital status, 41.9 % were single and 57.1 % were married (Table 1). As to the distribution of occupational characteristics, according to employment type, 89.9 % were regular workers, 7.2 % contract workers, and 2.9 % part-time/temporary workers (Table 1).

**Table 1:** Demographic and occupational characteristics of study population

Characteristics	Frequency (n)	Percentage (%)
Total	4,639	100.0
Age (years)		
Under 29	1,327	28.6
30 ~ 39	1,965	42.4
40 ~ 49	1,007	21.7
Over 50	340	7.3
Gender		
Female	1,090	23.5
Male	3,549	76.5
Education (years)		
Middle school (≤9)	211	4.5
High school (≤12)	1,896	40.9
College(≤16)	2,348	50.6
Graduate(16 <)	184	4.0
Marital status		
Single	1,945	41.9
Married	2,648	57.1
Divorced/Separated	46	1.0
Employment type		
Regular	4,172	89.9
Contract	335	7.2
Part-time/temporary	132	2.9
Working hour (/week)		
Under 40	168	3.6
40 ~ 43	2,022	43.7
44 ~ 47	664	14.3
48 ~ 55	1,004	21.6
Over 56	781	16.8
Job type		
White-collar	2,483	53.5
Blue-collar	2,156	46.5
Industry type		
Manufacturing	3,295	71.0
Non-manufacturing	1,344	29.0
Position		
Rank-and-file worker	2,959	63.8
Assistant manager/ deputy manager	956	20.6
Manager or higher	724	15.6
Industry size (person/site)		
Under 50	468	10.1
50 ~ 99	1,663	35.9
100 ~ 299	1,139	24.5
Over 300	1,369	29.5
Existence of break		
No	1,324	28.5
Yes	3,315	71.5

### Distribution of job stress according to general characteristics

We divided the subjects into 4 groups by assigning the total score of job stress obtained using the short-form questionnaire on job stress to the quartiles of the reference scale and analyzed according to demographic characteristics. In the results, according to age, the score of job stress was statistically significantly higher in the age group of 29 or younger than in the other age groups (*p* < 0.01), and according to gender, female showed a statistically significantly higher job stress score than male (*p* < 0.01). According to education level, those with low education level showed a higher percentage of those belonging to over 75 % of the reference scale, and according to marital status, the score of job stress was statistically significantly higher in the single group than in the divorced/separated group and the married group (*p* < 0.01) (Table 2).

**Table 2:** Distribution of reference scales of job stress according to demographic characteristics

Characteristics	Reference scales of job stress				*p*-value*
	Less than 25 %	25 % ~ 50 %	50 % ~ 75 %	More than 75 %	
Total (n = 4,639)	2,647(57.0)	918(19.8)	680(14.7)	394(8.5)	
Age (years)					
Under 29	763(57.5)	226(17.0)	214(16.1)	124(9.3)	0.01
30 ~ 39	1,088(55.4)	439(22.3)	273(13.9)	165(8.4)	
40 ~ 49	590(58.6)	195(19.4)	140(13.9)	82(8.1)	
Over 50	206(60.6)	58(17.0)	53(15.6)	23(6.8)	
Gender					
Female	698(64.0)	75(6.9)	204(18.7)	113(10.4)	<0.01
Male	1,949(54.9)	843(23.8)	476(13.4)	281(7.9)	
Education					
Middle school (= < 9)	98(46.4)	51(24.2)	37(17.5)	25(11.9)	<0.01
High school (= < 12)	983(51.9)	401(21.1)	327(17.2)	185(9.8)	
College (= < 16)	1,439(61.3)	430(18.3)	302(12.9)	177(7.5)	
Graduate (16 <)	127(69.0)	36(19.6)	14(7.6)	7(3.8)	
Marital status					
Single	1,087(55.9)	360(18.5)	295(15.2)	203(10.4)	<0.01
Married	1,538(58.1)	549(20.7)	373(14.1)	188(7.1)	
Divorced/Separated	22(47.8)	9(19.6)	12(26.1)	3(6.5)	

*: *p*-value was calculated by chi-square test for reference scale of job stress according to demographic characteristics

As to the distribution of job stress according to occupational characteristics, on the other hand, according to employment type, the percentage of those with high job stress was higher in part-time/temporary workers than in contract workers and regular workers, but the difference was not statistically significant. According to working hour, the percentage of those with high job stress was higher in those with longer working hour, and the difference was statistically significant (*p* < 0.01). According to job type, the percentage of those with high job stress was higher in blue-collar workers than in white-collar workers (*p* < 0.01), but no difference was observed according to industry type. In addition, the percentage of those with high job stress was higher in those of low position, those working at a large-size workplace, and those without breaks (*p* < 0.01) (Table 3).

**Table 3:** Distribution of reference scales of job stress by occupational characteristics

Characteristics	Reference score group				*p*-value*
	Less than 25 %	25 % ~ 50 %	50 % ~ 75 %	More than 75 %	
Total (*n* = 4,639)	2,647(57.0)	918(19.8)	680(14.7)	394(8.5)	
Employment type					
Regular	2,395(57.4)	823(19.7)	598(14.4)	356(8.5)	0.28
Contract	179(53.4)	71(21.2)	62(18.5)	23(6.9)	
Part-time/temporary	73(55.3)	24(18.2)	20(15.1)	15(11.4)	
Working hour (/week)					
Under 40	114(67.8)	24(14.3)	21(12.5)	9(5.4)	<0.01
40 ~ 43	1,252(61.9)	361(17.9)	265(13.1)	144(7.1)	
44 ~ 47	416(62.6)	121(18.2)	86(13.0)	41(6.2)	
48 ~ 55	508(50.6)	236(23.5)	166(16.5)	94(9.4)	
Over 56	357(45.7)	176(22.5)	142(18.2)	106(13.6)	
Job type^a^					
White-collar	1,630(65.7)	413(16.6)	286(11.5)	154(6.2)	<0.01
Blue-collar	1,017(47.2)	505(23.4)	394(18.3)	240(11.1)	
Industry type					
Manufacturing	1,781(54.1)	718(21.8)	489(14.8)	307(9.3)	0.1
Non-manufacturing	866(64.4)	200(14.9)	191(14.2)	87(6.5)	
Position					
Rank-and-file worker	1,576(53.2)	564(19.1)	505(17.1)	314(10.6)	<0.01
Assistant manager/ deputy manager	564(59.0)	207(21.6)	124(13.0)	61(6.4)	
Manager or higher	507(70.0)	147(20.3)	51(7.1)	19(2.6)	
Industry size (person/site)					
Under 50	267(57.1)	91(19.4)	78(16.7)	32(6.8)	<0.01
50 ~ 99	948(57.0)	354(21.3)	225(13.5)	136(8.2)	
100 ~ 299	600(52.7)	248(21.8)	187(16.4)	104(9.1)	
Over 300	832(60.8)	225(16.4)	190(13.9)	122(8.9)	
Existence of break					
No	702(53.0)	248(18.7)	233(17.6)	141(10.7)	<0.01
Yes	1,945(58.7)	670(20.2)	447(13.5)	253(7.6)	

*: *p*-value was calculated by chi-square test for reference scale of job stress according to occupational characteristics

^a^: job type: Blue-collar was all job related manufacture and white-collar was all other jobs

### Distribution of nicotine dependence

Of the subjects of this study, 1,948 current smokers were separated from non-smokers and ex-smokers, and nicotine dependence was analyzed for the current smokers. Of the current smokers, 17.9 % and 17.7 % got a nicotine dependence score of 2 and 3, respectively, and 0.3 % got a score of 10. When nicotine dependence was divided into 4 levels (low, medium, high and very high), 72.7 % of the current smokers belonged to the low level, 10.6 % to the medium level, 12.7 % to the high level, and 4.0 % to the very high level (Table 4).

**Table 4:** Distribution of total score and grade of nicotine dependence

Characteristics	Frequency (*n*)	Percentage (%)
Total score		
0	179	9.3
1	256	13.3
2	342	17.8
3	342	17.8
4	279	14.5
5	202	10.5
6	153	7.9
7	91	4.7
8	56	2.9
9	16	0.8
10	6	0.3
Grade^a^		
Low (0 ~ 5)	1,600	83.3
High (6 ~ 10)	321	16.7
Total	1,921	100

^a^: Grade: Low is under 5 of total score. High is over 6 of total score

### Relationship between job stress factors and nicotine dependence

Among job stress factors, interpersonal conflict, organization system and lack of reward were higher in the high dependence group (Table 5).

**Table 5:** Distribution of job stress score by nicotine dependence grade according to job stress factors

			Unit: mean [95 % Confidence interval]	
Job stress factors	Nicotine dependence grade		Total	*p*-value*
	Low	High		
Total [N (%)]	1,600(83.3)	321(16.7)	1,921(100.0)	
Job demand	49.8 [49.0, 50.6]	50.2 [48.6, 51.7]	49.9[49.2, 50.6]	0.74
Job control	49.3 [48.5, 50.0]	48.2 [46.3, 50.1]	49.1 [48.4, 49.8]	0.31
Interpersonal conflict	38.7 [38.0, 39.4]	40.7 [39.1, 42.3]	39.0 [38.4, 39.6]	0.02
Job insecurity	49.6 [49.0, 50.1]	49.8[48.5, 51.2]	49.6 [49.1, 50.1]	0.68
Organization system	46.6 [45.8, 47.3]	48.9 [47.2, 50.6]	46.9 [46.2, 47.6]	0.01
Lack of reward	45.8 [45.1, 46.6]	48.1 [46.1, 50.0]	46.2 [45.5, 46.9]	0.02
Occupational climate	36.7[36.0, 37.4]	36.5 [35.0, 38.1]	36.7 [36.0, 37.3]	0.83
Sum of 7 factors	45.1 [44.7, 45,5]	46.3 [45.4, 47.2]	45.3 [45.0, 45.7]	0.02

*: *p*-value was calculated by student T-test with nicotine dependence grade in job stress factors

### Relationship between the reference scale of each job stress factor and nicotine dependence

In the results of analyzing nicotine dependence according to the score of reference scale by job stress factor, significant difference was observed in job control(*p* < 0.05) and marginal statistical significance was found in interpersonal conflict and organization system (Table 6). Nicotine dependence was decreased in higher job control score and increased in higher organization system score, lack of reward score and total stress score according to multivariate logistic analysis (Table 7).

**Table 6:** Distribution of job stress scales by nicotine dependence grade with job stress factors divided by reference scales

Reference scales by job stress factors	Nicotine dependence grade		Total	*p*-value*
	Low	High		
Total	1,600(83.3)	321(16.7)	1,921(100.0)	
Job demand				
Less than 25 %	349 (21.8)	56 (17.4)	405 (21.1)	0.138
25 % ~50 %	635 (39.7)	144 (44.9)	779 (40.5)	
50 % ~75 %	296 (18.5)	65 (20.2)	361 (18.8)	
More than 75 %	320 (20.0)	56 (17.5)	376 (19.6)	
Job control				
Less than 25 %	385 (24.1)	99 (30.8)	485 (25.3)	0.016
25 % ~50 %	656 (41.0)	117 (36.4)	773 (40.2)	
50 % ~75 %	272 (17.0)	40 (12.5)	312 (16.2)	
More than 75 %	286 (17.9)	65 (20.3)	351 (18.3)	
Interpersonal conflict				
Less than 25 %	24 (1.5)	4 (1.2)	28 (1.5)	0.055
25 % ~50 %	1,027 (64.2)	181 (56.4)	1,208 (62.9)	
50 % ~75 %	283 (17.7)	69 (21.5)	352 (18.3)	
More than 75 %	266 (16.6)	67 (20.9)	333 (17.3)	
Job Insecurity				
Less than 25 %	291 (18.2)	58 (18.1)	349 (18.2)	0.985
25 % ~50 %	1,061 (66.3)	212 (66.0)	1,273 (66.3)	
50 % ~75 %	-	-	-	
More than 75 %	248 (15.5)	51 (15.9)	299 (15.5)	
Organization system				
Less than 25 %	564 (35.3)	93 (29.0)	657 (34.2)	0.059
25 % ~50 %	572 (35.7)	120 (37.4)	692 (36.0)	
50 % ~75 %	233 (14.6)	46 (14.3)	279 (14.5)	
More than 75 %	231 (14.4)	62 (19.3)	293 (15.3)	
Lack of reward				
Less than 25 %	694 (43.4)	124 (38.6)	818 (42.6)	0.237
25 % ~50 %	346 (21.6)	66 (20.6)	412 (21.4)	
50 % ~75 %	263 (16.4)	59 (18.4)	322 (16.8)	
More than 75 %	297 (18.6)	72 (22.4)	369 (19.2)	
Occupational climate				
Less than 25 %	939 (58.7)	191 (59.5)	1,130 (58.8)	0.935
25 % ~50 %	-	-	-	
50 % ~75 %	505 (31.6)	98 (30.5)	603 (31.4)	
More than 75 %	156 (9.7)	32 (10.0)	188 (9.8)	
Sum of 7 factors				
Less than 25 %	860 (53.7)	149 (46.4)	1,009 (52.5)	0.107
25 % ~50 %	401 (25.1)	91 (28.4)	492 (25.6)	
50 % ~75 %	202 (12.6)	46 (14.3)	248 (12.9)	
More than 75 %	137 (8.6)	35 (10.9)	172 (9.0)	

*: *p*-value was calculated by Chisq-test with nicotine dependence grade in reference scale of job stress factor

**Table 7:** Univariate and multivariate logistic analysis for nicotine dependence with job stress factors adjusted by general characteristics

Job stress factors	Univariate		Multivariate^a^	
	Crude Odds ratio	95 % Confidence interval	Adjusted Odds ratio	95 % Confidence interval
Job demand	1.00	[0.993, 1.009]	0.99	[0.978, 1.001]
Job control	0.99	[0.989, 1.004]	0.98	[0.969, 0.993]
Interpersonal conflict	1.01	[1.002, 1.018]	0.99	[0.986, 1.010]
Job insecurity	1.00	[0.992, 1.013]	0.99	[0.979, 1.006]
Organization system	1.01	[1.002, 1.018]	0.99	[0.983, 1.010]
Lack of reward	1.01	[1.001, 1.016]	0.99	[0.982, 1.008]
Occupational climate	0.99	[0.990, 1.008]	0.98	[0.970, 0.996]
Sum of 7 factors	1.02	[1.003, 1.032]	1.07	[1.015, 1.143]

^a^: adjusted by age, education, marital status, job-type

## Discussion

This study analyzed relationship between job stress and nicotine dependence, and is meaningful in that it demonstrated that job stress can be a problem in reducing smoking based on the finding that the smoking group showed high job stress and those with high job stress and subjective stress showed high nicotine dependence and, consequently, job stress was in a relationship with nicotine dependence.

In Korea, the smoking rate in 20-year-old or older adult men was 79.3 % in 1980 but decreased continuously down to 64.9 % until 1999. Then it maintained the level without a notable change but decreased sharply to 60.5 % in 2002, 50.3 % in 2005, and 20.7 % in 2010. Among 20-year-old or older adult women, on the contrary, the rate was 12.6 % in 1980 and decreased steadily down to 3.0 % until 2000, and then showed inconsistent up and down to 6.0 % in 2002, 4.0 % in 2004, and 3.1 % in 2005 [12]. After that, it showed a continuous decrease and reached 2.2 % in 2010. In our results, the overall smoking rate was 42.0 %, similar to 41.4 % reported as workers’ smoking rate by Kim et al. [13]. The age group of 30–39 showed the highest smoking rate as 47.3 %, suggesting that the smoking rate is relatively higher among young adults. In addition, men’s smoking rate was 54.1 %, somewhat higher than the report of Korean Institute for Health and Social Affairs, while women’s was 2.5 %, somewhat lower. According to smoking cessation attempt rate of Korean adults, on the other hand, 48.3 % of male smokers and 38.9 % of female ones had tried to stop smoking within the previous one year. In addition, the rate was higher in young smokers [14]. In our study, however, the percentage of ex-smokers among those who experienced smoking at least once in the past was 24.4 % (621/2542) of male smokers and 25 % (9/36) of female ones, showing a far lower smoking cessation attempt rate than before. This result was somewhat different from previous data in Korea.

And this study divided the subjects into the quartiles of job stress score and examined the groups according to demographic characteristics. The job stress level was higher in the young age groups than in the old age groups, in men than in women, in the lowly educated groups than in the highly educated groups, and in the divorced/separated group. Also in terms of occupational characteristics, job stress was higher in those working longer, in blue-collar workers than in white-collar workers, in the low position groups than in the high position groups, in the group without breaks than in the group having breaks during office hours. With regard to these relationships between occupational characteristics and stress, there was a report that the stress recognition rate was higher in manufacturing industry according to industry type, in temporary workers according to employment type, in white-collar workers according to job type, and in those working over 56 hours according to working hour [15].

Broms et al. [16] stated that marriage can reduce smoking through protective caring among the family members against nicotine dependence. In this study, however, nicotine dependence was not significantly different according to marital status. Schmidt et al. [17] reported that marital status did not affect nicotine dependence as found in this study but low education was a decisive risk factor for nicotine dependence. In addition, Finney et al. [18] argued that high-level education can reduce the risk factors of smoking by working as a protective factor on nicotine dependence. In this study as well, nicotine dependence was higher when education level was low. Furthermore, nicotine dependence was relatively higher in older groups, confirming indirectly that nicotine dependence grows stronger with increasing smoking period. The higher nicotine dependence in low education level groups and old groups may be related to the finding that subjective stress was high in these groups.

John et al. [19] investigated the correlation between nicotine dependence and job stress factors using FTND, and found that smoking was not associated with job stress. According to the report of Kouvonen et al. [20], however, imbalance between effort and reward can be a risk factor increasing smoking. In our study as well, nicotine dependence was higher in blue-collar workers according to job type, and in managers or higher-position workers according to position. What is more, Schmidt et al. [17] reported that excessive workload is rather related with low nicotine dependence. This is probably because workers having many breaks may smoke more using the breaks. In this study, working hour and whether to have breaks were not highly related with nicotine dependence, but the smoking rate was higher in those with longer working hour and those having many breaks and this suggests that the smoking rate goes down when workload is high.

This study was analyzed that the job stress factors were in a relationship with demographic and occupational characteristics. That is, nicotine dependence was higher in those whose score was high in organization system, and lack of reward. As the results of multivariate logistic analysis, high job control score effected nicotine dependence. Organization system and lack of reward score effected too negatively. Those stress factors were more effected Korean worker. This result may suggest that smokers keep smoking as a relief from job stress even if they recognize smoking to be a serious problem to their health. Therefore, job stress is believed to have an adverse effect on individuals and organizations as a risk factor inducing smoking or checking the fall of the smoking rate.

On the other hand, health promotion through smoking cessation in workplace can be efficient because the targets are specific and limited. Moreover, the consequent low turnover rate can bring not only health-related benefits to participating workers but also diverse benefits to the company including low absence rate, saving of medical expenses, high morale, low morbidity, and improved welfare as well as company image. Anti-smoking policies in workplace can reduce workers’ smoking quantity by inhibiting smoking during working hours and such efforts can be extended outside the workplace and reduce the number of regular smokers. In addition, the number of workers deciding to stop smoking is increasing as individual workers are increasingly aware of the harms of smoking and various studies have reported that smoking cessation can reduce damage to health. Nevertheless, it is hard to succeed in smoking cessation and therefore appropriate management is required for raising the smoking cessation success rate. Thus, the result of this study showing the correlation between job stress and smoking status and nicotine dependence can be used as an objective indicator for preventing smoking induced by job stress and for developing adequate smoking cessation strategies. Based on the results of this study, we may need to invent ways to enhance workers’ welfare for health promotion in workplace through improving working environment in order to reduce job stress and consequently to decrease smoking. What is more, because job stress is related not only to individuals’ health problems but also to industrial and social problems, it is urgently demanded to establish means and systems to control and reduce job stress. Moreover, as it was reported recently that stressful situations in workplace are related directly to the morbidity of various diseases and in particular chronic diseases [11, 21], the importance of job stress is expected to grow higher not only for workers but also for people’s practice of healthy life, chronic disease control and disease prevention, and therefore, it is considered essential to introduce ways for lowering the smoking rate through decreasing job stress.

## Conclusions

Job stress was found to be related with smoking status and nicotine dependence. Based on this result, it is suggested to enhance workers’ welfare for health promotion in workplace by improving their working environment in order to reduce job stress and consequently to decrease the smoking rate.
